# Tensile Bond Strengths of Two Adhesives on Irradiated and Nonirradiated Human Dentin

**DOI:** 10.1155/2015/798972

**Published:** 2015-12-10

**Authors:** Cécile Bernard, Cyril Villat, Hazem Abouelleil, Marie-Paule Gustin, Brigitte Grosgogeat

**Affiliations:** ^1^UFR d'Odontologie, Université Lyon 1, Université de Lyon, 11 rue Guillame Paradin, 69372 Lyon, France; ^2^Service de Consultations et Traitements Dentaires, Hospices Civils de Lyon, 6-8 Place Deperet, 69365 Lyon, France; ^3^Laboratoire des Multimatériaux et Interfaces, CNRS UMR 5615, Université Lyon 1, Université de Lyon, 11 rue Guillaume Paradin, 69372 Lyon, France; ^4^Département de Santé Publique, Institut des Sciences Pharmaceutiques et Biologiques (ISPB), EA4173, Université Lyon 1, Université de Lyon, 8 avenue Rockefeller, 69373 Lyon, France

## Abstract

The aim of this study was to assess the effect of radiotherapy on bond efficiency of two different adhesive systems using tensile bond strength test. Twenty extracted teeth after radiotherapy and twenty nonirradiated extracted teeth were used. The irradiation was applied *in vivo* to a minimal dose of 50 Gy. The specimens of each group were randomly assigned to two subgroups to test two different adhesive systems. A three-step/etch-and-rinse adhesive system (Optibond FL) and a two-steps/self-etch adhesive system (Optibond XTR) were used. Composite buildups were performed with a nanohybrid composite (Herculite XTR). All specimens were submitted to thermocycling ageing (10000 cycles). The specimens were sectioned in 1 mm^2^ sticks. Microtensile bond strength tests were measured. Nonparametric statistical analyses were performed due to nonnormality of data. Optibond XTR on irradiated and nonirradiated teeth did not show any significant differences. However, Optibond FL bond strength was more effective on nonirradiated teeth than on irradiated teeth. Within the limitations of an *in vitro* study, it can be concluded that radiotherapy had a significant detrimental effect on bond strength to human dentin. However, it seems that adhesive choice could be adapted to the substrata. According to the present study, the two-steps/self-etch (Optibond XTR) adhesive system tested could be more effective on irradiated dentin compared to three-steps/etch-and-rinse adhesive system (Optibond FL).

## 1. Introduction

“Radio-induced caries” are a well-known consequence of the radiotherapy of head and neck cancer malignant tumors. Hyposalivation which is induced by irradiation [1, 2], dietary changes [3], and oral flora modifications [4, 5] are considered as the most important etiological factors of these caries [6]. Radio-induced caries begin near the gum and surround the cervical zone of the tooth leading to coronoradicular fracture [7]. The loss of mechanical autocleaning of these surfaces as a result of decreased salivary flow probably explains this location.

While there is lack of data published on this topic, evidences suggest a conservative approach using adhesive restorations [8]. Haveman and Redding have shown that conventional glass-ionomer cement (GIC) had poorer results than the resin-modified glass-ionomer cements (RMGICs) and composite fillings in patients treated by radiotherapy [9]. Moreover, according to several studies, it is not recommended to use the GIC as restorative material for patients suffering hyposalivation and having a daily fluoride application [10–12]. Composite resin restorations are an alternative for both esthetic and wear resistance.

The loss of adhesive restorations can be due to an alteration of dental tissues as a consequence of head and neck irradiation. A significant decrease of dentin microhardness has been observed after irradiation [13]. These observations were accompanied by reduction of the stability of the enamel/dentin junction [14]. The disturbance of enamel/dentin junction could result in the formation of a gap (10 *μ*m), loss of prismatic structure, and bacterial colonization associated with the obliteration of the dentinal tubules and odontoblastic process atrophy [15, 16]. These characteristics can be observed via scanning electron microscopy (SEM) [17, 18]. Furthermore, the radiogenic destruction of the dentin collagen could result in bonding failure [19].

As the loss of these restorations is time dependent, it was suggested as a reliable method to test the durability of the bond strength by accelerated ageing [20–27]. Thermocycling tests evaluate the stress of adhesive interface to water infiltration, mechanical and contraction/expansion tension by an alternative immersion in cold water (5°C) and hot water (55°C) [28]. This can result in cracks which propagate along the adhesive interface, a process known under the name of “percolation” [29]. This method of ageing is suitable for dental adhesive systems and recommended by the International Organization for Standardization (ISO, TR 11450) [30].

The purpose of this study was to evaluate the incidence of the radiotherapy on tensile strength of two adhesives on the human irradiated and nonirradiated dentin.

## 2. Material and Methods

### 2.1. Sample Preparation

Forty human extracted teeth (incisors, canines, premolars, and molars) were collected (gathered following informed consent). Twenty came from irradiated patients suffering from head and neck cancer. These teeth received a minimal dose of 50 Gy and were extracted because of periodontal disease. Twenty other teeth came from nonirradiated patients and were used as control group. All teeth were collected and stored in physiological solution for a period not exceeding two weeks; then, they were stored in distilled water at a temperature of 5°C. Class I cavities on molars and class V cavities on other teeth (4 × 4 × 2 mm) were prepared with a cylindrical medium-grit (100 mm) diamond bur (FG 068-040, Komet France SA, Paris, France) under constant water irrigation. The burs were changed for every 8 teeth.

### 2.2. Experimental Design and Bonding Procedures

Each group was randomly divided into 2 subgroups of 10 teeth. The subgroups were restored using a two-step/self-etch adhesive system (Optibond XTR, batch number 5092152, Kerr France, Créteil, France) or a three-step/etch-and-rinse adhesive system (Optibond FL, batch number 4995918, Kerr France, Créteil, France). The adhesive materials were used following manufacturer's instructions (Table 1).

Restorations were made using a nanohybrid composite resin (Herculite XRV Ultra, Kerr France, Créteil, France) with 2 layers of 1 mm thickness. Photopolymerization of the resin-based materials was performed using a LED light curing unit (Elipar S10, 3M ESPE, Cergy-Pontoise, France) at 1450 mW/cm^2^.

Subsequently, the resin-bonded samples of each group underwent artificial ageing using thermocycling machine (10000 cycles for 2 weeks) with baths at temperatures of 5°C and 55°C (Table 2) and 30-second dwelling time. The storage solution of thermocycling baths was changed weekly.

### 2.3. Sticks Preparation

Thermocycled teeth were included in resin to allow fixation during microtensile sample preparation. Four to six slices, 1 mm thick, were cut perpendicularly and through to the bonded interface using Diamond Disk Wafering Blades 15HC (Buelher, Düsseldorf, Germany) under constant irrigation (IsoMet Low Speed Saw, Buelher, Düsseldorf, Germany). The sticks were then individualized and measured (±1 mm wide square section). The most peripheral sticks with residual enamel were excluded. A maximum of 4 sticks of the tooth central part were used trying to minimize the regional variability of dentin. The bonded surface area was calculated before each test by measuring the width with digital caliper.

### 2.4. Microtensile Bond Strength Testing (*μ*TBS)

Each specimen was attached following the methodology described by Perdigao et al. [31]. An aluminum device constituted of two symmetric parts, having a central notch (2 mm of depth and width) in order to allow autoalignment. Device surfaces were cleaned with alcohol. Tensile load was applied with a universal testing machine (DY34, Adamel Lhomargy SARL, Roissy-en-Brie, France), at a crosshead speed of 1 mm/min, to obtain the ultimate tensile strength, using a load cell of 1 KN.

### 2.5. Failure Mode Analysis

Fracture mode was determined at ×50 magnification with a stereoscopic microscope (Wild Heerbrugg TYP 376788, Wild Heerbrugg, Switzerland) and recorded as cohesive failure and adhesive failure.

### 2.6. Statistical Analysis

The experimental design included (i) two fixed crossed factors: irradiation [yes(I)/no(NI)] and adhesive system (XTR/FL) leading to 4 subgroups and (ii) a random factor (tooth) nested in each subgroup: 10 teeth per subgroup with one to four replicates per tooth. The conditions for the application of statistical treatment were carefully verified. The effect of the tooth factor on the explained variable (bond strength of sticks: *μ*TBS) was first assessed by a mixed linear model on the full dataset. In case of nonapplicability of this mixed model, we conducted a one-way nonparametric ANOVA per subgroup using Kruskal-Wallis test. Missing data were supposed to be missing at random and no data imputation was performed.

In case of no tooth effect on *μ*TBS, normality of *μ*TBS data was checked graphically and using the normality Shapiro test for each of the 4 subgroups. In case of nonrespect of normality in one subgroup, pairwise distributions comparisons were performed between subgroups. Four comparisons were* a priori* of interest: between the two control subgroups (NI:XTR versus NI:FL), between the two irradiated subgroups (I:XTR versus I:FL), and for each adhesive system: (NI:XTR versus I:XTR) and (NI:FL versus I:FL). Correction for multiple comparisons was performed to maintain the family-wise error rate at the significant level of 5%. For 4 pairwise comparisons, Bonferroni correction gave a significant level of 2-tailed single test equal to 0.05/4, that is, 0.0125. Data were reported as mean ± SD per subgroup. Statistics were performed using the R language, version 3.1.2 available on the https://cran.r-project.org/ website. Package nlme was used to perform mixed linear model.

## 3. Results

Three teeth and one tooth out of 10 were missing in NI:XTR and I:FL subgroups, respectively. Linear mixed model was not appropriate because of the nonnormality of the normalized residuals (*p* < 10^−3^). No effect of factor tooth was significant in each subgroup using Kruskal-Wallis test with *p* values ranging from 0.46 (I:XTR) to 0.84 (I:FL).

Due to the different number of samples by tooth, we obtained 15 observations for subgroup NI:XTR, 31 for NI:FL, 25 for I:XTR, and 27 for I:FL. The two subgroups relative to XTR exhibited nonnormal skewed distribution with *p* values < 10^−2^.

**Figure 1:**
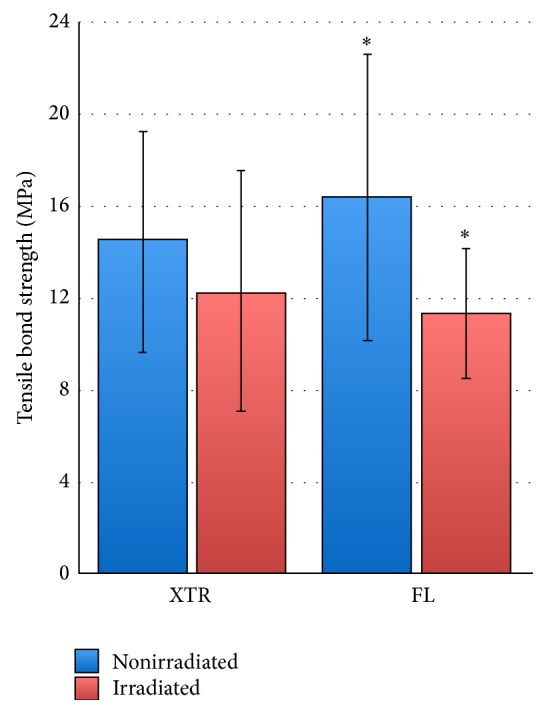
Mean of microtensile bond strength with standard deviation (MPa) according to the irradiation and the adhesive system (XTR/FL).  ^*∗*^Significant difference between results (*p* < 0.0125).

Means and standard deviation of *μ*TBS are graphically presented in box plots in Figure 1.

On irradiated dentin, both adhesive systems (XTR and FL) did not show any significant difference with *μ*TBS in I:XTR subgroup equal to 12.2 ± 5.3 MPa (mean ± SD) and in I:FL subgroup 11.3 ± 2.8 MPa (*p* = 0.97 > 0.0125). On nonirradiated dentin, they did not show any significant difference on bond strength with *μ*TBS in NI:XTR subgroup equal to 14.5 ± 4.8 MPa and in NI:FL subgroup 16.4 ± 6.2 MPa (*p* = 0.42 > 0.0125).

Regarding FL groups, the value was significantly different between nonirradiated and irradiated dentin (*p* = 0.0009 < 0.0125). *μ*TBS was observed 1.5 times higher in nonirradiated subgroup in case of FL (33% decrease from nonirradiated to irradiated subgroups). On the other hand, no statistical differences were found for XTR adhesive system (*p* = 0.040 > 0.0125) with *μ*TBS observed 1.2 times higher in nonirradiated subgroup (16% of decrease from nonirradiated to irradiated subgroups).

The failure type for each group is summarized in Table 3. Adhesive failures at the composite resin/dentin interface were mainly observed for specimens treated with XTR. For FL adhesive system, there were as many adhesive fractures as cohesive failure.

## 4. Discussion

Head and neck cancers are one of the most common cancers [32]. Surgery and/or radiotherapy are the treatment of choice for such cancers [33]. Among the adverse effects like xerostomia or osteoradionecrosis, it has been demonstrated by several authors that radiation affects hard tissues [13, 34–37]. Regarding these consequences, some studies evaluated the bond strength on irradiated teeth. However, thermocycling for a sufficient time was not considered [38, 39]; furthermore, teeth were irradiated outside the oral cavity and after extraction [39, 40].

The procedure of the present study considers both the use of* in vivo* irradiated teeth and a sufficient thermocycling ageing protocol.

Teeth were stored in physiological saline solution immediately after extraction at the dental clinic and then in distilled water at 5°C. Even though Goodis et al. noticed that the physiological salt solution could have an action on dentin permeability and on traction resistance, unlike distilled water [41], Retief et al. have shown that saline solution does not influence the chemical and physical properties of human dentin [42].

Cavities were prepared using diamond bur under continuous water cooling to bring a higher traction resistance, compared with the abrasive 80-grits paper and to the diamond bur without irrigation [43]. As the experimental conditions should be standardized, dental composite resins were bonded on flat surface despite the overestimated bonding strength resulting in comparison to clinical conditions [44].

Regional differences in dentin anatomy and permeability have a significant influence on dentin bond strength [45, 46].

Photopolymerization time was applied according to the manufacturer recommendations and using the same light curing unit [47]. For all groups, the same resin composite and the same shade were used to avoid any influence of the composite material on bonding [48]. Several studies have shown the influence of the thermocycling ageing on adhesive systems strength [23, 26]. The standard (ISO TR 11450) recommends 500 cycles [30]. To simulate one-year ageing, as in the study of Gale and Darvell, a 10000-cycle experiment has been performed [28].

Several studies involving the two adhesive systems used in this work have been performed and have shown similar results [26, 49, 50]. Furthermore, according to De Munck et al. meta-analysis [51], Optibond FL, is the current reference in term of dentin bonding efficiency, on all the adhesives. These studies have been made on normal dentin. Nevertheless, the results obtained in this study showing *μ*TBS decrease (33%) in irradiated dentin for FL subgroups are consistent with literature. It is reported that the ionizing radiations may have an effect on the collagen fibers of dentinal tubules [19, 52]. Moreover, the changes described in the crystalline structure of dental hard tissues after irradiation seem to affect tensile strength [53–56].

With the XTR adhesive system, the weak decrease of *μ*TBS (16%) in irradiated dentin could be due to the chemical connections between the carboxylic or the phosphate groups of functional monomers and the phases of dissolved hydroxyapatite. These chemical connections would contribute to a better cohesion of the infiltrated resin after polymerization and, probably, in better resistance in the hydrolysis of this zone [57].

The results are in agreement with those of Naves et al. and S. Yadav and H. Yadav [40, 57]. Nevertheless, another similar study [58] did not find significant differences between irradiated and nonirradiated groups according to four adhesive systems, taking in consideration that no process of artificial ageing has been applied. In the present study, teeth were irradiated* in vivo* and, then, underwent adverse effects like hyposalivation.

## 5. Conclusion

The changes resulting from the irradiation on the hardness, the crystalline structure or the collagen matrix, seem to influence the adhesive agents bond strength to dentin. The dental substrate might have experienced radiation effects that could compromise bonding ability by impairing hybrid layer formation.

Under the limitations of this* in vitro* study, it appears that, regarding the type of adhesive system, radiotherapy may affect the microtensile bond strength of composite restorations on irradiated dentin. Therefore, it is advisable for a clinician to restore all cavities before radiotherapy and initiate caries prevention modalities in patients undergoing radiation therapy.

Further studies are needed to help the practitioner to adapt the choice of the adhesive system after radiotherapy of head and neck.

## Figures and Tables

**Table 1:** Adhesive systems reference and composition.

Product name (manufacturer)	Class of adhesive	Composition	Batch number
Optibond FL, Kerr France, Créteil, France	3-step/etch-and-rinse adhesive	*Gel etchant*: 37.5% H_3_PO_4_, water, and fumed silica *Primer*: (Ph = 1.8): HEMA, GPDM, MMEP, water, ethanol, photoinitiator (CQ), and BHT *Adhesive*: Bis-GMA, HEMA, GPDM, GDMA, photoinitiator (CQ), ODmab, and fillers (fumed SiO_2_, barium aluminoborosilicate, and Na_2_SiF_6_)	4995918

Optibond XTR, Kerr France, Créteil, France	2-step/self-etch adhesive	*Primer*: (pH = 2.4 before application, reduction in 1.6 to the application in dental structure). Acetone, water, ethanol, HEMA, photoinitiator (CQ), and GPDM *Adhesive*: ethanol, HEMA, sodium hexafluorosilicate, MEHQ; nanosilica, barium; photoinitiator (CQ)	5092152

HEMA: 2-hydroxyethyl methacrylate.

GPDM: glycerol dimethacrylate dihydrogen phosphate.

MMEP: mono(2-methacryloyloxy)ethyl phthalate.

CQ: camphorquinone.

BHT: butylated hydroxytoluene.

Bis-GMA: bisphenol A glycidyl methacrylate.

GDMA: glycerol dimethacrylate.

MEHQ: monomethyl ether of hydroquinone.

**Table 2:** Different constituents and brands of the thermocycling machine.

Bath of hot water	Brand, Fisherbrand (water bath heated, digital PID control UK plug 12L)
Bath of cold water	Fisher (Bioblock Scientific 18201)

Waterproof box for electric system	Schneider Electric, Telemecanique crouzet (ACM)

Timer for arm	Crouzet (Top 948, LCD MULTI-FUNCTION TIMER)

**Table 3:** Adhesive and cohesive failure distribution.

	Adhesive fracture	Cohesive fracture
FL nonirradiated	48%	52%
FL irradiated	52%	48%
XTR nonirradiated	67%	33%
XTR irradiated	78%	22%
